# Lifestyle Assessment of Primary Healthcare Physicians in Taif, Saudi Arabia in the Year 2022

**DOI:** 10.7759/cureus.37323

**Published:** 2023-04-09

**Authors:** Abdullah S Alamri, Fawaz S Al-Otaibi, Ali O Alzahrani, Abdullah S Alharthi, Raed M Alfaran, Ahmed S Alzahrani

**Affiliations:** 1 Department of Preventive Medicine, Prince Mansour Military Hospital, Taif, SAU; 2 Department of Family Medicine, Alhada Armed Forces Hospital, Taif, SAU; 3 Department of Family Medicine, Prince Mansour Military Hospital, Taif, SAU

**Keywords:** taif city, saudi arabia, lifestyle, primary healthcare physicians, healthy lifestyles

## Abstract

Objective

This study aims to evaluate primary healthcare physicians’ lifestyles to promote their well-being and improve care quality for the general population.

Methods

This cross-sectional quantitative study was conducted on primary healthcare physicians in Taif, Kingdom of Saudi Arabia (KSA), using self-administered questionnaires.

Results

We included 206 participants aged 26-66. Most participants were 35 years old or younger (67%), male (62.1%), and residents (52.4%). Of all participants, 49.5% held a Bachelor’s degree, 40.8% had completed their board certificate or Ph.D., and 69.9% had at least 10 years of experience. Of all participants, 16.5% and less than 9% reported having hypercholesterolemia and other comorbidities, respectively. More than 50% were physically inactive, 26.2% were moderately inactive, and 17.4% were moderately active or active individuals. Physical activity was significantly associated with job titles (p < 0.018). The qualification was associated with dietary score (p = 0.034), and 42.7% of participants were in need of diet change. About a quarter (25.2%) were smokers, and 92.3% of them smoked daily. Male participants were associated with a greater likelihood of smoking (p < 0.001). Overall, 41.7% were overweight, and 25.7% were obese. Increased BMI was associated with older age and male gender (p < 0.001 and p < 0.002, respectively), as well as the title of the physician and years of experience (p < 0.001 and p < 0.002, respectively).

Conclusion

Participants’ unhealthy lifestyles indicate the need to establish measures to promote healthy lifestyles among physicians.

## Introduction

It is well-known that the health of physicians has an impact on their patients' well-being. Research has linked physicians' health behaviors with patient interactions [1]. Leading a healthy lifestyle is not only important for physicians' own well-being but also sets a positive example for their patients [2]. However, physicians who do not practice what they preach often face criticism from their patients and colleagues [3-5]. This is because physicians' behaviors are seen as indicators of harmful conduct [6]. Physicians who are not proactive in providing guidance for a healthier lifestyle may be less effective if they are not practicing it themselves [2].

The link between physicians' lifestyle choices and the care they provide patients is fundamental since primary healthcare physicians play an essential role in managing overweight and obesity by giving counseling and evidence-based advice to their patients concerning health risks associated with obesity. A literature review and meta-analysis showed that primary healthcare providers significantly and positively influence patients' attempts to change behaviors related to body weight [7]. However, another study carried out to investigate overweight and obesity among physicians in the Kingdom of Saudi Arabia (KSA) found a 36% and 23.2% prevalence of obesity and overweight, respectively, among resident physicians in the postgraduate training programs of the Saudi Board in the Aseer Region [8]. Moreover, it has been observed that overweight and obese physicians are significantly less keen than their normal-weight colleagues in counseling their patients on diet or exercise [9]. Overweight and obese physicians often believe that patients do not trust their advice regarding weight loss compared to normal-weight physicians, who can act as a model for weight-related behaviors [9].

This indicates how the quality of care to patients can be affected by a physician's own health status and lifestyle, such as smoking and exercise habits. In a recent Saudi study, the prevalence of current smoking among healthcare workers was 18.4%, while for ex-smoking, it was 9.8% [10]. Therefore, this would affect physicians' advice to patients. It has been reported that primary care physicians prefer to discuss smoking when patients have smoking-related problems [11], and patients with smoking-related morbidity are more likely to recall their physician's advice [11]. Patients view clinicians as a trusted and respected source of health advice regarding physical activity. Many patients regularly visit their doctors, allowing for continual and progressive health counseling opportunities about regular physical activity [12]. A recent study conducted on Australian general practitioners (GPs) found that the proportion with high knowledge and confidence in giving physical activity advice did not increase over the last seven years, despite several education initiatives conducted during this period [7]. This may be due to a lack of motivation for doctors to advise their patients, which might be explained by the high prevalence of obesity among physicians.

A study from Bahrain found that the mean BMI was 27.8±5 and that the most commonly reported health problems for primary healthcare physicians were hyperlipidemia (25.5%), hypertension (20.3%), and diabetes (11%). Almost a third performed at least 30 minutes of exercise weekly, and 13% exercised at least five days per week. The majority of them (98%) never had alcohol. Their average BMI was 27.8±5, and the prevalences of overweight and obesity were 39% and 33%, respectively [13].

On the other hand, a cross-sectional study included 322 Saudi physicians and found that most scored over 50% for healthy nutrition. However, the minority had a normal BMI (38.7%), 8.4% were smokers, and 21.1% did physical exercises [14]. Although a few researchers investigated this topic among Saudi physicians, the paucity still exists. Therefore, this study aims to assess primary healthcare physicians' lifestyles to promote the well-being of healthcare providers and the general population.

## Materials and methods

This is an analytical, cross-sectional study that utilized a self-administered questionnaire to collect data from participants. The study was conducted at primary healthcare centers (PHCCs) belonging to the Ministry of Health of Taif, KSA, and Family Medicine departments at military hospitals in Taif (Prince Mansour Military Hospital for Community Medicine, Alhada Hospital for Armed Forces, and Prince Sultan Military Hospital). The target population for this study consisted of all primary healthcare physicians of both genders, all nationalities and job titles, working at primary healthcare centers, belonging to the Ministry of Health in Taif (n = 177 physicians), and those working at military hospitals in Taif (n = 58 physicians). A consecutive sampling technique was employed as all subjects who met the criteria were invited, and a total of 206 respondents were included, accounting for approximately 88% of the target population.

Data collection technique and tool

The researcher met all the recruited physicians at their workplace and measured their weight and height. Then, every subject was asked to complete the self-administered questionnaire.

The current study utilized a self-administered questionnaire that included five main sections. The first section gathered demographic data of the physicians (age, gender, nationality, marital status, highest qualification, and job title), smoking history, and further smoking patterns. Weight and height were measured by the researcher and filled into the questionnaire. Obesity in adults was categorized according to the National Institute of Health: BMI greater than or equal to 30 kg/m² were considered obese, BMIs from 25 to 29.9 kg/m² were considered overweight, BMIs from 18.5 to 24.9 kg/m² were considered normal, and BMIs less than 18.5 kg/m² were considered underweight [15]. Physical exercise was assessed using a validated General Practice Physical Activity Questionnaire (GPPAQ), which was developed by the London School of Hygiene and Tropical Medicine as a validated short measure of physical activity to assess adult (16-74 years) physical activity levels [16]. The GPPAQ provides a simple four-level Physical Activity Index (PAI) assigning subjects to one of the following categories [17]: inactive (sedentary job and no physical exercise or cycling), moderately inactive (sedentary job and some [<1 hour] physical exercise and/or cycling per week or standing job and no physical exercise or cycling), moderately active (sedentary job and 1-2.9 hours physical exercise and/or cycling per week or standing job and some [<1 hour] physical exercise and/or cycling per week or physical job and no physical exercise or cycling), or active (sedentary job and ≥ 3 hours physical exercise and/or cycling per week, standing job and 1-2.9 hours physical exercise and/or cycling per week, physical job and some [<1 hour] physical exercise and/or cycling per week, or heavy manual job). ​​​​​​​ Nutrition assessment was conducted by the British Heart Foundation's "How healthy is your diet?" Questionnaire (2009) [18]. It consists of six main diet categories and 24 items. Each item has two possible answers (either "Yes" or "No"). Those who scored more than 12 (>50%) were categorized as needing to change their diet to be healthier.

Ethical issues

Institutional ethical approval was obtained from the King Abdulaziz City for Science and Technology Institutional Review Board (IRB Registration Number: HAP-02-T-067; approval number: 550). Participation was voluntary after obtaining approval from participants, and confidentiality was assured. The research was conducted in accordance with the Declaration of Helsinki.

Statistical analysis

The data were collected, coded, and cleaned. Data analysis was carried out using IBM SPSS Statistics for MacOS, version 27 (IBM Corp., Armonk, NY, USA). Descriptive statistics (i.e., number, percentage, median, and interquartile range) were used to summarize the data. Moreover, analytic statistics using the chi-square test (χ2) and the extension of the Fisher-Freeman-Halton Exact Test were applied, and p-values <0.05 were considered statistically significant.

## Results

A total of 206 individuals met the inclusion criteria and were included in the study analysis. Although participants' ages ranged from 26 to 66, most participants (67%) were 35 or younger. Male physicians represented the majority (62.1%) of the participants, and 52.4% of the total number had the title of a resident. Furthermore, 49.5% held a Bachelor's degree, while 40.8% have completed their board certificate or Ph.D. The included sample had a wide range of years of experience (1-40 years); however, 69.9% of them had ≤10 years in the field (Table 1). The study participants were asked if they had been diagnosed with any chronic disease, and 16.5% of the participating individuals reported a diagnosis of hypercholesterolemia. Meanwhile, the prevalence of other comorbidities was below 9% among the study sample (Table 2).

**Table 1:** Demographic data (n=206).

Characteristics	Frequency	%
Age group		
≤35	138	67.0
36-45	47	22.8
46-55	9	4.4
≥56	12	5.8
Gender		
Male	128	62.1
Female	78	37.9
Job title		
Resident	108	52.4
Registrar	12	5.8
Senior registrar	58	28.2
Consultant	28	13.6
Qualification		
Bachelor's degree	102	49.5
Diploma/Masters	20	9.7
Board certified/Ph.D.	84	40.8
Working years		
≤10	144	69.9
11-20	41	19.9
21-30	9	4.4
≥31	12	5.8

**Table 2:** Prevalence of comorbidities among participants (n=206).

Comorbidity	Frequency	%
Diabetes mellitus	16	7.8
Hypertension	18	8.7
Hypercholesterolemia	34	16.5
Ischemic heart disease	2	1.0

The current study sheds light on smoking status and patterns among the participating physicians. Table 3 shows that 25.2% of the included individuals are currently smoking tobacco, and 92.3% of them have the habit of smoking tobacco on a daily basis. The group who reported positive current smoking tobacco use status have a median of 10 years of smoking with an IQR of 10 years. Furthermore, the median number of cigarettes they smoke daily is 12, with an IQR of 15.

**Table 3:** Smoking habits among participants (n=206).

Smoking habits	Frequency	%
Tobacco use (current)		
No	154	74.8
Yes	52	25.2
Do you smoke tobacco daily		
No	4	46.7
Yes	48	92.3
How many years have you been smoking cigarettes? median (Interquartile range)	10 (10)	
How many cigarettes do you smoke per day? median (Interquartile range)	12 (15)	

Moreover, the present study assesses the lifestyle of the participating physicians through four main outcomes: BMI, dietary score, physical activity level, and current tobacco use. Furthermore, the study investigated the associated factors with each of these outcomes. In regards to BMI, 41.7% were overweight, 32.5% were normal weight, and 25.7% were obese. Several factors showed statistical significance in association with BMI, such as age and gender (p < 0.001 and p < 0.002, respectively). Moreover, qualification, title, and years of experience were statistically associated with BMI, and these associations were significant (p < 0.001, p < 0.002, and p < 0.001, respectively). Among the chronic diseases, only hypercholesterolemia showed a statistically significant association with the participants' BMI (p < 0.0001) (Table 4).

**Table 4:** Associated factors with BMI among participants (n=206). Chi-square Test, *Fisher-Freeman-Halton Exact Test.

	Normal weight		Overweight		Obese		
Factors	N	%	N	%	N	%	P-value
Age (years)							
≤35	58	86.6	48	55.8	32	60.4	<0.001*
36-45	9	13.4	27	31.4	11	20.8	
46-55	0	0.0	4	4.7	5	9.4	
≥56	0	0.0	7	8.1	5	9.4	
Gender							
Male	31	46.3	64	74.4	33	62.3	0.002
Female	36	53.7	22	25.6	20	37.7	
Qualification							
Bachelor's degree	46	68.7	39	45.3	17	32.1	<0.001
Diploma/Masters	0	0.0	13	15.1	7	13.2	
Board certified/Ph.D.	21	31.3	34	39.5	29	54.7	
Job title							
Resident	46	68.7	43	50.0	19	35.8	0.002
Registrar	0	0.0	9	10.5	3	5.7	
Senior registrar	16	23.9	20	23.3	22	41.5	
Consultant	5	7.5	14	16.3	9	17.0	
Working years							
≤10	60	89.6	49	57.0	35	66.0	<0.001*
11-20	7	10.4	26	30.2	8	15.1	
21-30	0	0.0	4	4.7	5	9.4	
≥31	0	0.0	7	8.1	5	9.4	
Diabetes mellitus							
No	65	97.0	77	89.5	48	90.6	0.200
Yes	2	3.0	9	10.5	5	9.4	
Hypercholesterolemia							
No	66	98.5	72	83.7	34	64.2	<0.001
Yes	1	1.5	14	16.3	19	35.8	
Hypertension							
No	64	95.5	78	90.7	46	86.8	0.24
Yes	3	4.5	8	9.3	7	13.2	
Ischemic heart disease							
No	67	100.0	86	100.0	51	96.2	0.65*
Yes	0	0.0	0	0.0	2	3.8	
Total	67	32.5	86	41.7	53	25.7	

Table 5 shows the overall status of dietary score among the participants, in addition to the results of investigating the association between demographic variables and dietary score. In general, 42.7% of the participants needed a dietary change in their diet according to their dietary score. Although multiple factors were studied for the association, few showed statistically significant association. The physicians' qualifications were associated with the participants' dietary scores, and this association achieved statistical significance (p = 0.034). In addition, the study found a statistically significant association with the physicians' title, that is, resident, consultant, etc. (p < 0.001).

**Table 5:** Factors associated with dietary score among participants (n=206). Chi-square Test, *Fisher-Freeman-Halton Exact Test.

	No changes needed		Need to change diet		
Factors	N	%	N	%	P-value
Age (years)					
≤35	80	67.8	58	65.9	0.290
36-45	26	22.0	21	23.9	
46-55	3	2.5	6	6.8	
≥56	9	7.6	3	3.4	
Gender					
Male	70	59.3	58	65.9	0.335
Female	48	40.7	30	34.1	
Qualification					
Bachelor's degree	62	52.5	40	45.5	0.034
Diploma/Master	6	5.1	14	15.9	
Board certified/Ph.D.	50	42.4	34	38.6	
Job title					
Resident	62	52.5	46	52.3	<0.001
Registrar	6	5.1	6	6.8	
Senior registrar	22	18.6	36	40.9	
Consultant	28	23.7	0	0.0	
Working years					
≤10	84	71.2	60	68.2	0.269
11-20	22	18.6	19	21.6	
21-30	3	2.5	6	6.8	
≥31	9	7.6	3	3.4	
Diabetes mellitus					
No	108	91.5	82	93.2	0.660
Yes	10	8.5	6	6.8	
Hypercholesterolemia					
No	102	86.4	70	79.5	0.187
Yes	16	13.6	18	20.5	
Hypertension					
No	108	91.5	80	90.9	0.877
Yes	10	8.5	8	9.1	
Ischemic heart disease					
No	118	100.0	86	97.7	0.180*
Yes	0	0.0	2	2.3	
Total	118	57.3	88	42.7	

The study attempted to measure the physical activity level and identify the factors associated with different physical activity levels among the included sample of physicians. In the current study sample, more than 50% of the participants were physically inactive, and 26.2% were moderately inactive. On the other hand, 17.4% of the total number of physicians were considered moderately active or active individuals. A physician's job title showed an association with their physical activity level, and this association was statistically significant (p = 0.018). Nevertheless, three out of four comorbidities were associated significantly with the physical activity level. Having a diagnosis of hypercholesterolemia, hypertension, or ischemic heart disease was statistically associated with the level of physical activity in the included sample (p = 0.04, .001, and 0.03, respectively) (Table 6).

**Table 6:** Associated factors with physical activity level among participants (n=206). Fisher-Freeman-Halton Exact Test.

	Inactive		Moderately inactive		Moderately active		Active		
Factors	N	%	N	%	N	%	N	%	P-value
Age (years)									
≤35	75	64.7	39	72.2	18	64.3	6	75.0	0.263
36-45	30	25.9	9	16.7	8	28.6	0	0.0	
46-55	5	4.3	4	7.4	0	0.0	0	0.0	
≥56	6	5.2	2	3.7	2	7.1	2	25.0	
Gender									
Male	66	56.9	36	66.7	20	71.4	6	75.0	0.364
Female	50	43.1	18	33.3	8	28.6	2	25.0	
Qualification									
Bachelor's degree	54	46.6	28	51.9	14	50.0	6	75.0	0.841
Diploma/Master	14	12.1	4	7.4	2	7.1	0	0.0	
Board certified/Ph.D.	48	41.4	22	40.7	12	42.9	2	25.0	
Job title									
Resident	56	48.3	30	55.6	16	57.1	6	75.0	0.018
Registrar	8	6.9	0	0.0	4	14.3	0	0.0	
Senior registrar	32	27.6	18	33.3	8	28.6	0	0.0	
Consultant	20	17.2	6	11.1	0	0.0	2	25.0	
Working years									
≤10	80	69.0	39	72.2	19	67.9	6	75.0	0.422
11-20	25	21.6	9	16.7	7	25.0	0	0.0	
21-30	5	4.3	4	7.4	0	0.0	0	0.0	
≥31	6	5.2	2	3.7	2	7.1	2	25.0	
Diabetes mellitus									
No	110	94.8	46	85.2	26	92.9	8	100.0	0.171
Yes	6	5.2	8	14.8	2	7.1	0	0.0	
Hypercholesterolemia									
No	94	81.0	50	92.6	20	71.4	8	100.0	0.04
Yes	22	19.0	4	7.4	8	28.6	0	0.0	
Hypertension									
No	98	84.5	54	100.0	28	100.0	8	100.0	0.001
Yes	18	15.5	0	0.0	0	0.0	0	0.0	
Ischemic heart disease									
No	116	100.0	54	100.0	26	92.9	8	100.0	0.03
Yes	0	0.0	0	0.0	2	7.1	0	0.0	
Total	116	56.3	54	26.2	28	13.6	8	3.8	

In addition to individual BMI, dietary score, and physical activity level, the study investigated tobacco use as one of the lifestyle domains. Only 25.2% of the participating physicians were smoking during data collection. Although all demographic data and chronic diseases were investigated for their association with tobacco use, only one variable achieved a statistically significant association. Apparently, gender was an associated factor with participants' tobacco use in the current study (p < 0.001) (Table 7).

**Table 7:** Associated factors with tobacco use among participants (n=206). Chi-square Test, *Fisher-Freeman-Halton Exact Test.

	No tobacco use		Tobacco use		
Factors	N	%	N	%	P-value
Age (years)					
≤35	98	63.6	40	76.9	0.203*
36-45	37	24.0	10	19.2	
46-55	9	5.8	0	0.0	
≥56	10	6.5	2	3.8	
Gender					
Male	80	51.9	48	92.3	<0.001
Female	74	48.1	4	7.7	
Qualification					
Bachelor's degree	78	50.6	24	46.2	0.130
Diploma/Master	18	11.7	2	3.8	
Board certified/Ph.D.	58	37.7	26	50.0	
Job title					
Resident	84	54.5	24	46.2	0.314*
Registrar	10	6.5	2	3.8	
Senior registrar	38	24.7	20	38.5	
Consultant	22	14.3	6	11.5	
Working years					
≤10	102	66.2	42	80.8	0.162*
11-20	33	21.4	8	15.4	
21-30	9	5.8	0	0.0	
≥31	10	6.5	2	3.8	
Diabetes mellitus					
No	140	90.9	50	96.2	0.371*
Yes	14	9.1	2	3.8	
Hypercholesterolemia					
No	128	83.1	44	84.6	0.833*
Yes	26	16.9	8	15.4	
Hypertension					
No	144	93.5	44	84.6	0.082
Yes	10	6.5	8	15.4	
Ischemic Heart Disease					
No	152	98.7	52	100.0	1.000*
Yes	2	1.3	0	0.0	
Total	154	74.8	52	25.2	

## Discussion

Our study assessed the primary healthcare physicians' lifestyles to promote the well-being of the healthcare providers and the general population. Through contact with the public, primary healthcare physicians act as models to the public to promote healthier lifestyles, thus preventing most non-communicable diseases, mainly lifestyle-related conditions that are an increasingly heavy burden to the Saudi healthcare system. This study will inform health officials and healthcare providers about their measures to promote healthy lifestyles among healthcare staff and improve care quality for the public.

This study's findings indicated that hypercholesterolemia was the most common chronic disease, and the prevalence of other comorbidities was below 9%. Hypercholesterolemia is a disorder characterized by high levels of low-density lipoprotein (LDL). This causes atherosclerosis, which increases one's risk of heart attack, stroke, and cardiovascular diseases, the leading causes of death worldwide [19]. In Saudi Arabia, 44% of people with chronic diseases have high blood pressure, 40% have type 2 diabetes, 21% have heart disease, and 9% have asthma [20]. Physicians with unhealthy lifestyles are at increased risk of chronic diseases, premature death, and high BMI. Most physicians could aggravate their risks, leading to unhealthy care providers and, eventually, poor quality of care. Studies have demonstrated that hypercholesterolemia is associated with weight gain and cardiovascular diseases due to atherosclerosis [21]. This might explain the prevalence of high BMI and hypercholesterolemia among our study participants. Another study conducted in Iran found a positive correlation between BMI and serum cholesterol level and found that obese people had a higher risk of hypercholesterolemia in early middle age than overweight people [22].

Another significant risk factor for heart disease is smoking due to the increased risk of atherosclerosis induced by cigarette chemical inhalation [23]. Smoking also causes other health issues like cancers, lung diseases, infections, etc. We found that around a quarter of physicians smoked, and half were daily smokers. This is less than the 34.8% reported by a study conducted in Riyadh. The study found that smoking was significantly enhanced by having a smoking family member or friend, being employed as a resident, having a medical specialty, and frequently being on call. Social stress and withdrawal symptoms were the most frequent triggers of relapse [24]. Smoking among physicians is a peculiar problem because they understand the risks and harm related to smoking. This might be due to high-stress levels at work since stress was associated with increased risks of smoking [25]. Our study showed that male physicians were significantly more likely to smoke than female physicians, in keeping with previous studies linking the male gender with an increased risk of smoking [26, 27].

We found associations between BMI and age and BMI and gender. BMI increased with age, and more males were overweight and obese than females. These findings substantiate studies indicating that the male gender and older age are associated with obesity [28].

Gender-specific behavioral differences, the popularity of fitness among women, and the media's widespread promotion of the "perfect" figure might explain why fewer women had high BMI than men.

Previous literature has identified other factors associated with high BMI: lower education levels, lower income, and unemployment [28]. In contrast, our study findings showed that higher degree levels were associated with lower BMI, and less work experience was associated with higher BMI. This may be because physicians with lower degree levels are mostly still in training or have tight schedules, increasing stress and preventing them from achieving healthy lifestyles by exercising and preparing healthy meals. This is confirmed by over half of the study's participants being residents and by the finding that diet change was needed in significantly more physicians with low degree levels and residents. The more experienced the physicians become, the less the burden of their responsibilities.

Our study showed that more than 50% of the participants were physically inactive, and most comorbidities were associated significantly with physical activity levels. These findings align with previous studies showing that physicians are less active than the general population and that such inactivity increases risks for chronic diseases [6, 13, 29]. It was found that most physicians spend a long time sitting, and lack of time and heavy work burden are the biggest obstacles to exercise and physical activity [29,30]. This study is limited by the cross-sectional design, which cannot identify causes and can only establish associations. Therefore, extensive prospective longitudinal research is recommended.

## Conclusions

This study showed that most primary healthcare physicians were inactive with high BMI. High BMI correlated with age and was more prevalent among male physicians. The prevalence of smoking was higher, mainly among residents and physicians with lower degree levels, despite their understanding of the risks. In spite of unhealthy lifestyles, chronic diseases were relatively less prevalent, with hypercholesterolemia being the most common.

These findings highlight the need for establishing measures to promote healthy lifestyles among physicians. Recommendations include revising schedules to ease working conditions so physicians have enough rest, educating physicians about the importance of healthy lifestyles in improving care quality, and establishing support groups.
